# Supplementary material for: Lack of evidence for associative learning in pea plants
Source: eLife. 2020 Jun 23;9:e57614. doi: 10.7554/eLife.57614 (PMC7311169; doi:10.7554/eLife.57614)
Supplement: Transparent reporting form [file elife-57614-transrepform.docx]

***eLife’s* transparent reporting form**

We encourage authors to provide detailed information *within their submission* to facilitate the interpretation and replication of experiments. Authors can upload supporting documentation to indicate the use of appropriate reporting guidelines for health-related research (see [EQUATOR Network](http://www.equator-network.org/%20)), life science research (see the [BioSharing Information Resource](https://biosharing.org/)), or the [ARRIVE guidelines](http://www.plosbiology.org/article/info:doi/10.1371/journal.pbio.1000412) for reporting work involving animal research. Where applicable, authors should refer to any relevant reporting standards documents in this form.

If you have any questions, please consult our Journal Policies and/or contact us: [editorial@elifesciences.org](mailto:editorial@elifesciences.org).

**Sample-size estimation**

- You should state whether an appropriate sample size was computed when the study was being designed
- You should state the statistical method of sample size computation and any required assumptions
- If no explicit power analysis was used, you should describe how you decided what sample (replicate) size (number) to use

Please outline where this information can be found within the submission (e.g., sections or figure legends), or explain why this information doesn’t apply to your submission:

No formal sample-size estimation was used. Informally, the study was designed with the largest sample size possible, specifically chosen to detect an effect much weaker than the original report. All data are reported in the Supplemental Data spreadsheet.

**Replicates**

- You should report how often each experiment was performed
- You should include a definition of biological versus technical replication
- The data obtained should be provided and sufficient information should be provided to indicate the number of independent biological and/or technical replicates
- If you encountered any outliers, you should describe how these were handled
- Criteria for exclusion/inclusion of data should be clearly stated
- High-throughput sequence data should be uploaded before submission, with a private link for reviewers provided (these are available from both GEO and ArrayExpress)

Please outline where this information can be found within the submission (e.g., sections or figure legends), or explain why this information doesn’t apply to your submission:

The primary set of experiments were repeated over four separate several-day periods, with data collection August 11, August 17, August 27, and September 3, 2017 (data are dated in Supplemental Data spreadsheet). Pilot experiments described in Methods were performed on two separate periods, but the first was cancelled due to technical problems with performance of the with data collection August 6.

Each plant was located in an individual Y maze with individual lights and fans, and were treated as biological replicates. There were no technical replicates. Data scoring protocol is summarized in. Exclusion criteria are stated in Methods. No outliers were observed.

**Statistical reporting**

- Statistical analysis methods should be described and justified
- Raw data should be presented in figures whenever informative to do so (typically when N per group is less than 10)
- For each experiment, you should identify the statistical tests used, exact values of N, definitions of center, methods of multiple test correction, and dispersion and precision measures (e.g., mean, median, SD, SEM, confidence intervals; and, for the major substantive results, a measure of effect size (e.g., Pearson's r, Cohen's d)
- Report exact p-values wherever possible alongside the summary statistics and 95% confidence intervals. These should be reported for all key questions and not only when the p-value is less than 0.05.

Please outline where this information can be found within the submission (e.g., sections or figure legends), or explain why this information doesn’t apply to your submission:

Statistical analysis followed Gagliano *et al*., main statistics are in Results, additional statistical analysis is available in Supplemental Data. The nature of the data precluded the generation of several of the statistical summaries requested – if there are any suggested methods to add such statistics, they will be added in Results.

(For large datasets, or papers with a very large number of statistical tests, you may upload a single table file with tests, Ns, etc., with reference to sections in the manuscript.)

**Group allocation**

- Indicate how samples were allocated into experimental groups (in the case of clinical studies, please specify allocation to treatment method); if randomization was used, please also state if restricted randomization was applied
- Indicate if masking was used during group allocation, data collection and/or data analysis

Please outline where this information can be found within the submission (e.g., sections or figure legends), or explain why this information doesn’t apply to your submission:

Group allocation was randomized using a random number generator, as described in Methods. As a rule, groups were assigned as late as possible, after potential sources of bias (always after the addition of the Y maze, in some cases after the training period).

Masking and blinding were used where possible. Y-mazes carried unique ID numbers, which were assigned randomly to plants when the pot-Y-maze assembly was created during each replication of the experiment. ID number was random with respect to position within the chamber and experimental group. After each ‘testing day’ when plants were to be scored, lights and fans were removed and plants were arranged in numerical order along a lab bench. After undergoing training on how to score plants, scorers were left with a notebook to record the results (L, R, N) of each plant with the experimenter out of the room. The experimenter input the raw data into spreadsheets, then mapped plant ID numbers back to experimental group to perform statistical analysis.

**Additional data files (“source data”)**

- We encourage you to upload relevant additional data files, such as numerical data that are represented as a graph in a figure, or as a summary table
- Where provided, these should be in the most useful format, and they can be uploaded as “Source data” files linked to a main figure or table
- Include model definition files including the full list of parameters used
- Include code used for data analysis (e.g., R, MatLab)
- Avoid stating that data files are “available upon request”

Please indicate the figures or tables for which source data files have been provided:

Data sufficient to generate all tables and figures is available in Supplemental Data spreadsheet.
